# Plants and Lactic Acid Bacteria Combination for New Antimicrobial and Antioxidant Properties Product Development in a Sustainable Manner

**DOI:** 10.3390/foods9040433

**Published:** 2020-04-04

**Authors:** Elena Bartkiene, Vita Lele, Vytaute Starkute, Paulina Zavistanaviciute, Egle Zokaityte, Ieva Varinauskaite, Greta Pileckaite, Laura Paskeviciute, Gintare Rutkauskaite, Tomas Kanaporis, Laura Dmitrijeva, Pranas Viskelis, Antonello Santini, Modestas Ruzauskas

**Affiliations:** 1 Department of Food Safety and Quality, Faculty of Veterinary, Lithuanian University of Health Sciences, Mickeviciaus str. 9, LT-44307 Kaunas, Lithuania; vita.lele@lsmuni.lt (V.L.); vytaute.starkute@lsmuni.lt (V.S.); paulina.zavistanaviciute@lsmuni.lt (P.Z.); egle.zokaityte@lsmuni.lt (E.Z.); ieva.varinauskaite@stud.lsmu.lt (I.V.); greta.pileckaite@stud.lsmu.lt (G.P.); laura.paskeviciute@stud.lsmu.lt (L.P.); tomas.kanaporis@stud.lsmu.lt (T.K.); laura.dmitrijeva@stud.lsmu.lt (L.D.); 2 Institute of Animal Rearing Technologies, Faculty of Animal Sciences, Lithuanian University of Health Sciences, Mickeviciaus str. 9, LT-44307 Kaunas, Lithuania; 3 Institute of Horticulture, Lithuanian Research Centre for Agriculture and Forestry, Kauno str. 30, LT-54333 Babtai, Lithuania; biochem@lsdi.lt; 4 Department of Pharmacy, University of Napoli Federico II, Via D. Montesano 49, 80131 Napoli, Italy; 5 Institute of Microbiology and Virology, Faculty of Veterinary, Lithuanian University of Health Sciences, Mickeviciaus str. 9, LT-44307 Kaunas, Lithuania; modestas.ruzauskas@lsmuni.lt; 6 Department of Anatomy and Physiology, Faculty of Veterinary, Lithuanian University of Health Sciences, Mickeviciaus str. 9, LT-44307 Kaunas, Lithuania

**Keywords:** nutraceuticals, by-products, antimicrobial ingredients, essential oil, lactic acid bacteria, blackcurrants

## Abstract

In this study, nutraceuticals based on antimicrobial ingredients (*Artemisia absinthium* water extract and essential oil (EO), *Lactobacillus uvarum* LUHS245 strain cultivated in a whey media, and blackcurrants juice (BCJ) preparation by-products were developed. In addition, two texture forming agents for nutraceutical preparations were tested (gelatin and agar). The developed nutraceutical ingredients showed antimicrobial properties: *Artemisia absinthium* EO (concentration 0.1%) inhibited methicillin-resistant *Staphylococcus aureus*, *Enterococcus faecium*, *Bacillus cereus*, *Streptococcus mutans*, *Staphylococcus epidermidis*, and *Pasteurella multocida*; LUHS245 strain inhibited 14 from the 15 tested pathogenic strains; and BCP inhibited 13 from the 15 tested pathogenic strains. The best formulation consisted of the *Artemisia absinthium* EO, LUHS245, and BCP immobilised in agar and this formulation showed higher TPC content (by 2.1% higher), as well as higher overall acceptability (by 17.7% higher), compared with the formulation prepared using gelatin.

## 1. Introduction

The global nutraceutical market has grown in the last few years and this growth is expected to continue. For this reason, the challenges related to high prices for nutraceutical compounds became very real [1]. One of the solutions to reduce prices, as well as ensure sustainability of the nutraceuticals preparation, is the valorization of the food processing industry by-products by recovering the functional compounds to be used for nutraceuticals preparation. Nowadays, industrial principles and sustainable innovations aiming at fully recycling by-products (BY), in which BY are used as raw materials for developing new ingredients and products, became very important [2,3,4,5,6]. The large amount of functional and bioactive materials loss in the BY that are usually treated as waste to dispose of and, in addition, the disposal of the food industry BY generates serious concerns regarding management, economic, and environmental problems [2]. Nonetheless, many of these BY can be valorized into other production systems, e.g. nutraceuticals preparation [3,4,5,6,7,8]. 

Cheese whey is a good example of a dairy industry BY that can be used and given high added value in nutraceutical formulations. Many treatments (biological, physicochemical, direct land application, thermal, precipitation, flocculation, acid precipitation, electrochemical, and membrane technologies) have been suggested for whey valorisation [9]. Nonetheless, roughly 50% of whey goes to disposal since it is not processed or valorized [10]. The main challenge to face in whey valorization is its components concentration [11]. From this point of view, very high value functional compounds could be projected to valorize whey substrate in an economical way. Whey retains up to 55% compounds from milk, however its composition is not stable and is varied; but, the beneficial characteristics of whey could be applied in food/nutraceuticals/feed industries [12]. At the same time, whey can be used to increase functional microorganisms viability during storage [13,14,15]. 

Other high quantities of the food industry BY are produced by juice preparation, and these BY are not enough and efficiently valorized. Today, consumers are interested in higher value foods, besides possessing sensory attractiveness and nutrients, to have health-promoting characteristics. It was published that a diet rich in berries/fruits positively affects human health, as well as regular consumption of berries/fruits may delay ageing processes and reduce the risk of various diseases [16,17,18]. Berry fruits have been identified as important sources of gallic and ellagic acid, as well as flavonoids, tannins, stilbenoids, and phenolic acids [16]. Also, berry and fruits polyphenols may act as antimicrobial agents for a wide range of pathogens [19]. Blackcurrants contain desirable phytochemicals, which possess antioxidant properties [20]. Blackcurrants polyphenols possess preventive and therapeutic effects [21], but a very high content of these valuable compounds are lost because they remain in the BY. 

Also, adoption as active substances sources from non conventionally used plants, such as *A. absinthium*, which is well known in traditional medicine, is nowadays becoming very popular. The *Artemisia essential* oils (EOs) contained high concentrations of myrcene, trans-thujone, and trans-sabinyl acetate. Abovementioned compounds inhibite *Candida albicans*, *Staphylococcus aureus*, *Trichophyton rubrum*, *Microsporum canis*, *Cryptococcus neoformans*, *Microsporum gypseum*, *Escherichia coli*, *Staphylococcus epidermidis*, *Fonsecaea pedrosoi*, and *Aspergillus niger* [22].

The end point of the proposed work was based on the fact that high value products preparation can be realised in a sustainable manner by using food industry BY valorization. In this experiment, nutraceuticals in chewing tablets form, based on antimicrobial ingredients (*Artemisia absinthium* extracts, *Lactobacillus uvarum* LUHS245 strain, and blackcurrants juice preparation BY), were developed. In addition, two texture forming agents for nutraceutical preparations were tested (agar and gelatin) and the best formulations according to quality parameters (color coordinates, texture, total phenolic compounds content, antioxidant activity, as well as sensory properties) were selected.

## 2. Materials and Methods 

### 2.1. Materials Used for Nutraceuticals Preparation

*Artemisia absinthium* essential oil (EO) was purchased from the Institute of Aromatherapy (Kaunas, Lithuania). *Artemisia absinthium* dried plant (underground part) was obtained from the local pharmaceutical company “Camelia” (Kaunas, Lithuania). The water extract was prepared by mixing 40.00 g of *Artemisia absinthium* dried plant with 1260 mL of boiled water (98 ± 2 °C temperature). After 30 min, the plant and water mixture was filtered through a gauze filter and used for antimicrobial analysis. Blackcurrants (*Ribes nigrum*, variety ”Ben Alder”) BY were obtained from the Institute of Horticulture, Lithuanian Research Centre for Agriculture and Forestry (Babtai, Kaunas distr., Lithuania) in 2018. Blackcurrants BY were dried by lyophilization (at −40 ± 2 °C). For lyophilization, a sublimator 3 × 4 × 5 (ZIRBUS Technology GmbH, Germany) was used. The condenser temperature was −85 °C and the vacuum was 2 × 10^−6^ MPa. The samples were frozen at −40 ± 2 °C in a laboratory freezer and then left in a freeze dryer for 72 h. Whey (lactose 4.0%, protein 0.8%, lactic acid 0.5%, pH value 4.45, titratable acidity 0.22, minerals 0.6%, total solids 6.5%) was purchased from JSC “Pieno zvaigzdes” (Kaunas, Lithuania) and used as a media for a *Lactobacillus uvarum* LUHS245 strain biomass growth. The experiment used LAB strain *Lactobacillus uvarum* LUHS245, which previously demonstrated good antimicrobial activities against a variety of pathogenic and opportunistic microorganisms, which was provided by Lithuanian University of Health Sciences collection (Kaunas, Lithuania). The LUHS245 strain was stored at −80 °C in a Microbank system (Pro-Lab Diagnostics, Birkenhead, UK) and grown in MRS broth (CM 0359, Oxoid, Hampshire, UK) at 30 °C for 48 h prior to use. The agar powder (*Gelidium sesquipedale* algae, Rapunzel, Germany) was used as a polymer with mucoadhesive properties for nutraceuticals forming. In addition, gelatin was also tested (Klingai, Lithuania).

### 2.2. Grown of the Lactobacillus uvarum LUHS245 in Whey Media

For *Lactobacillus uvarum* LUHS245 biomass growth, whey media was used and for growth parameters optimisation glucose (GLU), yeast extract (Y) and saccharose (SA) additives were tested. Prior to fermentation, the whey was sterilised, cooled to 30 °C, and different concentrations of Y, GLU, and SA (0.5; 1.0; 1.5; 2.0; 2.5; 3.0; 3.5; 4.0; 4.5; 5.0%) were added, with the purpose of improving the growth of the LUHS245. The optimal quantities of the Y, GLU, and SA, as well as the optimal fermentation time (the LAB count in the whey media was analysed every 3 h), were selected. Lyophilisation of the LUHS245 cultivated in the dairy production BY was performed in a sublimator 3 × 4 × 5 (ZIRBUS Technology GmbH, Harz, Germany). The parameters of the process were the same as described for blackcurrants BY lyophilisation. The LAB count in the lyophilised powder was determined. 

### 2.3. Lactic Acid Bacteria Count in Fermented Lyophilised Dairy Production By-Products Media

The lactic acid bacteria (LAB) count in fermented lyophilised dairy production BY was evaluated according to ISO 15214:1998 [23]. The petri plates, with sterile MRS agar (CM0361, Oxoid) of 5 mm of thickness, were separately seeded with the LAB diluted suspension using sowing in surface and were incubated under anaerobic conditions at 30 °C for 72 h. The number of LAB colonies on the surface of the MRS agar plate was calculated and expressed as log_10_ of colony forming units per gram (CFU g^−1^). 

### 2.4. Antimicrobial Activity of the Artemisia Absinthium Water Extract and Essential Oil, Lactobacillus uvarum LUHS245, and Blackcurrants By-Products Evaluation

Antimicrobial activity of the *Artemisia absinthium* water extract and essential oil (EO), *Lactobacillus uvarum* LUHS245, and blackcurrants BY against *Klebsiella pneumoniae*, *Salmonella enterica*, *Pseudomonas aeruginosa*, *Acinetobacter baumanni*, *Proteus mirabilis*, methicillin-resistant *Staphylococcus aureus* (*MRSA*), *Enterococcus faecalis*, *Enterococcus faecium*, *Bacillus cereus*, *Streptococcus mutans*, *Enterobacter cloacae*, *Citrobacter freundii*, *Staphylococcus epidermidis*, *Staphylococcus haemolyticus*, *Pasteurella multocida* was assessed by measuring the diameters of inhibition zones (DIZ, mm) in agar well diffusion assays. The abovementioned method was described in detail by Bartkiene et al. [24]. 

LUHS245 was grown in de Man Rogosa Sharpe (MRS) medium (Biolife, Milan, Italy) at 30 °C. Two percent of the MRS solution (*v*/*v*), in which the strain was cultured, were inoculated into fresh medium and propagated for 18 h. The cells were harvested by centrifugation (6000× *g*, 10 min, 4 °C). The culture supernatant was filtered through a 0.2 mm sterile Millipore filter (Merck KGaA, Darmstadt, Germany) to remove all cells and adjusted to pH 6.5 with 5 mol L^−1^ NaOH to eliminate the organic acids effect. Supernatants were used for the determination of the antimicrobial activities of LUHS245 strain against a variety of pathogenic and opportunistic bacterial strains. Agar well diffusion assay was used for the antimicrobial activities testing of the *Artemisia absinthium* water extract, LUHS245, and blackcurrants juice preparation by-products. For this purpose, 0.5 McFarland Unit density suspension of each pathogenic bacteria strain was inoculated onto the surface of cooled Mueller Hinton Agar (Oxoid, UK) using sterile cotton swabs. Wells of 6 mm in diameter were punched in agar and filled with 50 µL of the tested compounds. The antimicrobial activities against the tested bacteria were determined by measuring the diameter of the inhibition zones (mm). The experiments were repeated three times and the average of the inhibition zones was calculated. 

In addition, the antimicrobial activity of the *Artemisia absinthium* water extracts and EO against the abovementioned pathogenic and opportunistic strains were determined in liquid media [25]. Antimicrobial activity was defined as the *Artemisia absinthium* water extract (10%, 20%, and 30% concentrations) and/or EO (0.1% concentration) inhibiting visible microbial growth. For this purpose, water extracts and EO were placed into 1 mL of Mueller Hinton Broth (Oxoid, UK). Then, 10 µL of 0.5 McFarland density of the pathogenic and opportunistic bacterial strain, initially cultivated on Mueller Hinton Agar (Oxoid, UK), was added, mixed, and incubated at 35 °C for 24 h. After incubation, the viable pathogenic and opportunistic bacterial strains were controlled by plating them on an appropriate universal medium (Tryptic Soy Agar, Oxoid). The results were interpreted as (−) if the pathogens did not grow on universal medium and (+) if the pathogens grew on universal medium. Growth control without extracts and EO was carried out.

Experiments were performed in triplicate. 

### 2.5. Nutraceuticals Formulation

**Scheme 1:**
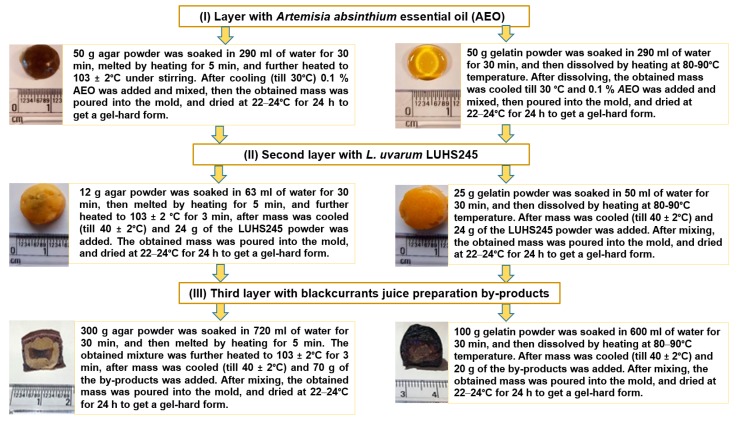
The main ingredients and formulation of the separate layer of the nutraceutical based on *Artemisia absinthium* EO, *Lactobacillus uvarum* LUHS245 cultivated in whey substrate, and blackcurrant juice preparation by-products.

The main ingredients, formulations, and technological steps of the separate layers of the nutraceuticals based on *Artemisia absinthium* EO, *Lactobacillus uvarum* LUHS245 cultivated in a whey media, and blackcurrant juice preparation BY, as well as the whole nutraceutical-product, are shown in Scheme 1. Two formulations of the three layer nutraceuticals based on agar and gelatin were prepared.

### 2.6. The Evaluation of Total Phenolic Compounds (TPC) Content in Separate Layers and the Whole Formulation of the Prepared Nutraceuticals and Their Antioxidant Activity 

The TPC content in separate layers and the whole formulation of the prepared nutraceuticals was determined by the spectrophotometric method, as reported elsewhere [26]. The absorbance of samples was measured at 765 nm using spectrophotometer J.P. SELECTA S.A. V-1100D (Barcelona, Spain). Antioxidant activity of the separate layers and the whole formulation of the prepared nutraceuticals was evaluated according to the method reported by Zhu et al. [27].

### 2.7. Evaluation of Nutraceuticals Colour Coordinates, Texture, and Overall Acceptability

The color characteristics were evaluated using a CIE *L*∗*a*∗*b*∗ (*L*∗ = lightness; *a*∗ = redness or −*a*∗ = greenness; *b*∗ = yellowness or −*b*∗ = blueness) system (CromaMeter CR-400, Konica Minolta, Japan). The hardness parameter was evaluated using a Brookfield texture analyser (Middleboro, MA, USA). Samples were compressed to 10% of their original height at a crosshead speed of 10 mm/s. The resulting peak force of compression was reported as nutraceutical hardness. Three replicates from three different sets of preparation were analyzed and averaged. The overall acceptability was carried out by 50 judges according to International Standards Organisation 8586-1 method [28], using a 140 mm hedonic line scale, ranging from 140 (extremely like) to 0 (extremely dislike).

### 2.8. Statistical Analysis

Statistical analysis was performed using multivariate analysis of variance (ANOVA). For data processing, software packages STATISTICA 7.1 (StatSoft Inc., Tulsa, USA) and SPSS 20.0 (IBM Corp., Armonk, NY, USA) were used. Statistical significance was set at *p* ≤ 0.05.

## 3. Results and Discussion

### 3.1. Antimicrobial Properties of the Artemisia Absinthium Water Extract and Essential Oil (EO), Lactobacillus uvarum LUHS245, and Blackcurrant Juice Preparation By-Products 

Inhibition of the growth of pathogenic bacteria by *Artemisia absinthium* water extract, *L. uvarum* LUHS245, and blackcurrant juice preparation BY is shown in Table 1. *Artemisia absinthium* water extract showed antimicrobial activity against *Pasteurella multocida* (20.4 ± 4.1 mm). *Lactobacillus uvarum* LUHS245 strain inhibited 14 from the 15 tested pathogenic strains, and the highest inhibition zones against *Pasteurella multocida* and *Bacillus cereus* 18 01 were found (22.0 ± 0.2 and 21.5 ± 0.3 mm, respectively). Blackcurrant juice preparation BY inhibited 13 from the 15 tested pathogenic strains, and the highest inhibition zones against *Pasteurella multocida*, *Bacillus cereus* 18 01, and *Streptococcus mutans* were found (30.7 ± 0.5, 21.4 ± 0.2, and 20.1 ± 0.1 mm, respectively). According to the results obtained, preparation of the nutraceuticals outer layer by using blackcurrant juice preparation BY can be very promising as a dental caries prophylaxis because antimicrobial activity against *Streptococcus mutans* was established. *Streptococcus mutans* can cause dental caries and it has been reported that more than 90% of the population is suffering from it [29].

Antimicrobial activities of the *Artemisia absinthium* EO (concentration 0.1%) and *Artemisia absinthium* water extract (concentrations 10%, 20%, and 30%) against pathogenic opportunistic microorganisms in liquid medium are shown in Table 2. *Artemisia absinthium* water extract does not inhibit pathogenic opportunistic microorganisms in liquid medium, however *Artemisia absinthium* EO (concentration 0.1%) inhibited methicillin-resistant *Staphylococcus aureus* M87fox, *Enterococcus faecium* 103, *Bacillus cereus* 18 01, *Streptococcus mutans*, *Staphylococcus epidermidis*, and *Pasteurella multocida*. According to the results obtained, for nutraceuticals preparation, *Artemisia absinthium* EO was selected. 

Natural plant ingredients are usually characterized as less toxic compared to synthetic ones [30]. Nowaday, EOs, as potential alternatives to synthetic antimicrobials, have become very popular [31]. It was published that terpenes are predominant compounds of the Artemisia absinthium EO [32] and trans sabinyl acetate is a predominant compound in the A. absinthium EO [32]. The lipophilicity of monoterpenes and sesquiterpenes were indicated as the main antimicrobial mechanism of action of the A. absinthium [33]. This characteristic leads to diffusion through microorganism membranes and inhibition of the synthesis processes in bacterial cells [34]. Earlier reports suggested that A. absinthium possesses antibacterial properties [35,36]. Also, it was published that the extracts inhibit pathogenic bacteria, but not LAB [37,38].

However, in our study, it was established that the *Artemisia absinthium* water extract inhibits the growth of *Pasteurella multocida* (inhibition zone 20.0 ± 4.1 mm), as well as EO (concentration of EO 0.1%) inhibited Methicillin-resistant *Staphylococcus aureus* M87fox, *Enterococcus faecium* 103, *Bacillus cereus* 18 01, *Staphylococcus epidermidis*, and *Pasteurella multocida*, Table 2; Table 3). According to the obtained results, EO, as a higher antimicrobial activity component, in comparison with *Artemisia absinthium* water extract, was selected for nutraceuticals preparation. 

The second layer of the nutraceuticals was prepared by using lyophilised in a whey media LUHS245 strain. LUHS245 strain showed antimicrobial activities against most of the tested pathogenic opportunistic strains (except *Streptococcus mutans*), and the highest inhibition zones against *Enterococcus faecium* 103, *Bacillus cereus* 18 01, *Staphylococcus epidermis*, and *Pasteurella multocida* were found (20.0 ± 0.5, 21.5 ± 0.3, 19.5 ± 0.4, and 22.0 ± 0.2 mm, respectively). Lactic acid bacteria are relevant for many aspects of health [39,40]. These bacteria are popular due to the several characteristics related to human/animal health [41]. LAB properties to inhibit pathogens in most of the cases are associated with the production of many metabolites because neutralization of the supernatant reduces antimicrobial properties, and this indicates that the most important factor remains low acidity of the fermentable substrate [42]. Natural antimicrobial agents are of great interest for applications in food, as well as the health industry [43]. LAB have a GRAS (Generally Recognized as Safe) status [44] and from this point of view, are meeting consumer preferences. The third layer of nutraceuticals was prepared by using blackcurrant juice preparation BY, and their antimicrobial activity is shown in Table 2. Blackcurrant juice preparation BY inhibited most of the tested pathogenic strains (except *Klebsiella pneumoniae* and *Enterobacter cloacae*), and the highest inhibition zones against *Pasteurella multocida* and *Bacillus cereus* 18 01 were found (30.7 ± 0.5 and 21.4 ± 0.2 mm, respectively). Blackcurrants are the richest sources of polyphenols of the berry group [45] and they reduce oxidative stress [46], which is a proinflammatory factor that plays a key role in the genesis of many serious diseases, including the civilizational ones, like circulatory diseases, tumors, or diabetes [47]. Bioactive phytocompounds are extra-nutritional constituents that typically occur in small quantities in foods [48], they vary widely in chemical structure and function, and have antioxidant, antiproliferative, antimicrobial, anti-diabetic, estrogenic, acetyl cholinesterase, anti-inflammatory activities, etc. [49,50]. Blackcurrants anthocyanins are one type of flavonoid phytopigments, which showed potential health benefits [51,52]. The localization of flavonoids/anthocyanins and their interaction with cell membranes plays an important role in membrane-mediated signaling cascades and influences anti-tumor [53,54], antimicrobial [55], and antioxidant [56,57] properties.

### 3.2. Grown and Stabilization of the Lactobacillus uvarum LUHS245 Strain in Dairy Production By-Products Media

**Figure 1:**
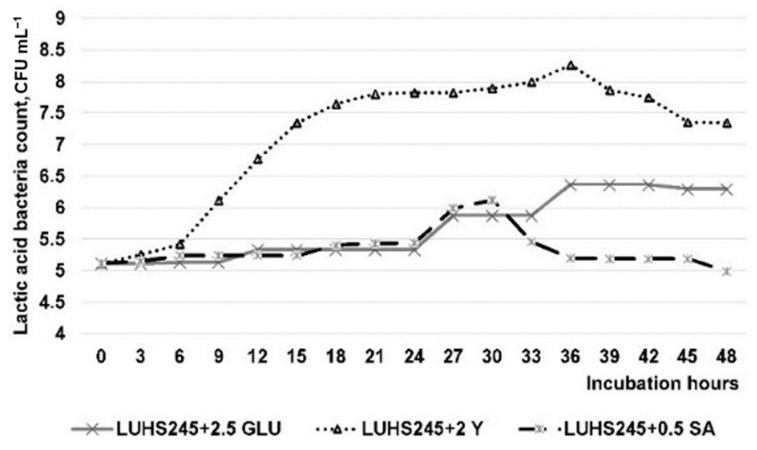
*Lactobacillus uvarum* LUHS245 biomass growth in dairy by-products media enriched with the optimum concentration of selected additives (LUHS245+2.5 GLU—whey enriched with 2.5% glucose and fermented with LUHS245; LUHS245+2 YE—whey enriched with 2.0% yeast extract and fermented with LUHS245; LUHS245+0.5 SACC—whey enriched with 0.5% sucrose and fermented with LUHS245).

Lactic acid bacteria growth after 12, 24, and 48 h of cultivation in whey (WHE) is presented in Table 3. After 12 h of incubation, the highest count of LAB was found in WHE with 4.0% of the yeast extract (Y) (~7.9 log_10_ CFU mL^−1^). After 24 h of incubation, the LAB count in the non-enriched WHE was reduced by 15.0%. Opposite tendencies were found in most of the WHE with glucose (GLU), Y, and saccharose (SA), and in many cases, the additives had a positive influence on the LAB growth. The highest LAB count was found in WHE with 2.0% of the Y (8.11 ± 0.14 log_10_ CFU mL^−1^). After 48 h of incubation, the LAB count was the same or decreased, except in samples enriched with SA, and the highest LAB count was found in WHE with 0.5% of SA (8.11 ± 0.13 log_10_ CFU mL^−1^). The LAB count was significantly influenced by the additives and their quantity, as well as fermentation time (*p* ≤ 0.0001), and the interaction of the analysed factors was significant (*p* ≤ 0.0001). The optimal quantities of the additives were 2.5% of GLU, 2.0% of Y, and 0.5% of SA. The results of the LAB growth in WHE with the optimum concentration of selected additives are presented in Figure 1. The optimal time for LUHS245 incubation in the WHE with 2.5% GLU, 2.0% Y, and 0.5% SA is 28 h. The highest LAB count in WHE with 2.0% of Y was observed after 36 h of incubation. The addition of GLU and SA had a lower influence on the growth of LAB, compared with the addition of Y. LUHS245 strain was incubated in the WHE with 2.5% GLU, 2.0% Y, and 0.5% SA for 28 h (optimal incubation time) and lyophilised. After lyophilisation, a viable LAB count in the obtained powder was 8.90 ± 0.12 log_10_ CFU g^−1^. 

The uses of technologically functionalized (e.g., dehydrated) starter cultures is more attractive, due to higher technological microorganisms survival and viability under environmental conditions [58]. Viability of LAB increasing for convenient industrial uses can be performed in a sustainable manner. From an environmental, as well as economical point of view, public awareness for food waste and by-products recycling has recently increased, and the dairy industry generates huge amounts of by-products [59,60]. Also, LAB biomass preparation by using by-products as nutrient media can lead to a profitable process [61]. Finally, whey is perspective sustainable media for LAB cultivation, as well as dehydratation.

Lyophilisation is the most popular process used to preserve LAB because of its ability to protect from spoilage, as well as its long viability during storage [62,63]. During freezing, bacterial cells are exposed to mechanical stress because of intra- and extra-cellular ice crystal formation and increased osmotic pressure caused by solutes in the remaining unfrozen fraction. This process can lead to the destruction of bacterial membranes and cause harmful damage [64]. Moreover, during lyophilisation, the removal of water by sublimation additionally increases osmotic pressure and can be harmful for membranes and surface proteins [65]. For this reason, cryprotectants are usually added to maintain the viability of microorganisms [64]. Different polymers, sugars, milk, honey, polyols, and amino acids have been tested for their protective effect during lyophilisation [64,65,66]. Disaccharides, such as maltose, sucrose, and trehalose, are able to induce shrinkage of the cells by osmosis-derived dehydration before freezing, thereby reducing intracellular ice formation [64]. Compounds mixing with different protective mechanisms can have a positive influence on the higher protection of microorganisms during freezing, as well as drying compared with single-component application, due to components’ synergic protective effects [64]. Skim milk, containing a mixture of lactoalbumine and casein, as well as saccharides, is a selected cryoprotectant for many LAB due to its ability to prevent cellular damage by stabilizing the cell membrane and providing a protein-protective coating for the cells [67]. Moreover, different sugars have high levels of protection of LAB during freeze-drying, due to their ability to replace structural water in membranes after dehydration [68]. It was reported that skim milk powder with lactose or saccharide contained the best cryoprotectans for lactobacilli [67]. For instance, *L. lactis* CIDCA 8221 lyophilised using milk and sucrose as a cryoprotective medium had a high survival rate and recovery ability [69]. Different additives can improve the viability of LAB in different matrices, as well as dehydratation processes, and increases the stability of strains, thus alleviating production on an industrial scale.

### 3.3. The Total Phenolic Compounds Content and Radical Scavenging Activity of Nutraceuticals 

The total phenolic compounds (TPC) content and antioxidant activity of the different layers, as well as whole nutraceutical, are given in Table 4. When comparing *Artemisia absinthium* EO+agar (Aex+Ag) and *Artemisia absinthium*+gelatin (Aex+G) layers, higher TPC content and antioxidant activity by the (Aex+G) layer was found (by 5.1 and 14.6% higher, respectively). Different tendencies of the second layer antioxidant properties were obtained, as a higher TPC content of the layer prepared with gelatin was found (by 4.8% higher), however higher antioxidant activity of the layer prepared with agar was established (by 7.2% higher). In comparison to whole nutraceutical formulations, higher TPC content was shown by products prepared with gelatin (by 2.1% higher), however antioxidant activity of the gelatin and agar prepared nutraceuticals was similar (with gelatin prepared, it was 75.15 ± 3.9% and with agar prepared, 81.96 ± 4.5%). Significant influence of the gelatin/agar uses on the antioxidant properties of the nutraceuticals was not established. 

Free radicals are very reactive in biological systems and an overproduction can lead to various life threatening disorders like atherosclerosis, inflammatory diseases, and cancer [69]. *A. absinthium* extract possesses antioxidant and free radical scavenging activities in vitro, as well as showing cytotoxic and antitumor activity against different malignancies [69]. Finally, *A. absinthium* extracts can be novel active drug compounds, as they have antiproliferative, antioxidant, and radical scavenging properties, however additional studies need to be undertaken to carry out the in vivo pharmacological experimentation of this plant [22]. Antioxidants, molecules with a radical scavenging capacity, are considered to exercise a defensive consequence against free radical injury and these molecular components may prevent many chronic diseases like cancer, hepatitis, asthma, atherosclerosis, arthritis, heart disease, and diabetes [70,71]. Currently, many synthetic antioxidants are also used, however these antioxidants have been thought to damage the liver and cause cancer [72]. Due to side effects and toxicity to health, their utilization is restricted [73]. The consumption of plant based additives as antioxidants increases day by day [72]. Plant based antioxidant demand continues to increase in the market, as they are used in food, cosmetic, and pharmaceutical industries to replace synthetic antioxidants. For this reason, plant based by-products can be a valuable source for these substances. Among natural antioxidants, phenolic substances have been of special interest because they are found extensively in plants [72]. Natural products containing phenolic substances have antioxidant potential, mostly because of their redox ability, which enables them to behave as hydrogen donors, single oxygen quenchers, and reducing agents as well as they have the potential to chelate metals [72]. The phenolic substances have taken part in body cell protection from harm by H_2_O_2_, lipid peroxide, unsaturated fatty acid, neutralizing, and absorbing free radicals [74]. However, it is not just plants based antioxidants that are promising for nutraceuticals formulations, the antioxidant properties of LAB were also published [75]. Finally, compositions were developed from different origins of antioxidant properties, showing that compounds can be very attractive for nutraceuticals formulations, as well as can lead to dose reducing and other activities, such as antimicrobial, increasing. 

### 3.4. Nutraceuticals Colour Coordinates, Texture, and Overall Acceptability

Nutraceuticals contain different biologically and chemically active compounds. Various processes are applied for nutraceutical ingredients technological functionalization. In this study, all the compounds were analysed in accordance with their antimicrobial properties, however especially plant compounds can possess antioxidant properties as well. However, for the product’s success in the market, it is not enough to provide only the desirable functional properties; colour, texture, and its related overall acceptability are also very important. Nutraceuticals colour coordinates, texture, and overall acceptability are shown in Table 4. When comparing *Artemisia absinthium* EO mmobilized in agar and gelatin, gelatin increased the lightness and yellowness of the first layer (by 58.0 and 68.7%, respectively), however agar or gelatin selection was not significant on the redness coordinates. When comparing the colour coordinates of the second layer, in all the cases, the layer prepared with gelatin showed higher lightness (by 9.8% higher), redness (by 83.6% higher), and yellowness (by 17.7% higher). Gelatin or agar selection for the outer layer preparation was not significant on the lightness and yellowness coordinates, however higher redness by using agar was found (by 8.6% higher).

When comparing the hardness of the texture, significant differences of the first layer texture hardness were not established, however harder texture of the second and outer layers by using agar was found (by 23.3% and 81.8%, respectively, higher). When comparing the overall acceptability of the separate layers, as well as whole formulation, it can be stated that the blackcurrant juice preparation by-products are very promising antimicrobial ingredients that are acceptable for consumers and can mask other ingredients for which acceptability is not very high. Between colour coordinates (lightness, redness, yellowness) and overall acceptability of the prepared nutraceuticals, negative moderate (*r* = −0.6632), positive very strong (*r* = −0.9001), and negative moderate (*r* = −0.6514) correlations were found. When comparing whole nutraceuticals formulation prepared with agar and gelatin, higher overall acceptability of the nutraceuticals prepared with agar was established (by 17.7% higher), however for between nutraceuticals texture and overall acceptability, a very low positive correlation (*r* = 0.2271) was established.

## 4. Conclusions

It can be summarized that antimicrobial and antioxidant properties showing nutraceuticals formulation in chewing tablets form, based on *Artemisia absinthium* EO (concentration ≤ 0.1 inhibited methicillin-resistant *Staphylococcus aureus* M87fox, *Enterococcus faecium* 103, *Bacillus cereus* 18 01, *Streptococcus mutans, Staphylococcus epidermidis*, and *Pasteurella multocida*), *Lactobacillus uvarum* LUHS245 encapsulated in a whey media (inhibited 14 from the 15 tested pathogenic strains), and blackcurrant juice preparation by-products (inhibited 13 from the 15 tested pathogenic strains) can be prepared in a sustainable manner. The best formulation consists of the *Artemisia absinthium* EO, *Lactobacillus uvarum* LUHS245 encapsulated in a whey media, and blackcurrant juice preparation by-products immobilized in agar as this formulation showed higher TPC content (by 2.1% higher), as well as higher overall acceptability (by 17.7% higher), compared with the formulation prepared with gelatin. Finally, compositions were developed from different origin antioxidant properties, showing that compounds can be very attractive for nutraceuticals formulations, as well as can lead to the dose reducing and other activities increasing, e.g. antimicrobial activity. Further experiments are planned to evaluate the antimicrobial activities of *Artemisia absinthium* extracts and LUHS245 strain against non-pathogenic bacterial strains with the aim to evaluate their influence on normal microbiota.

## Figures and Tables

**Table 1:** Inhibition of the growth of pathogenic bacteria by *Artemisia absinthium* water extract, *L. uvarum* LUHS245 cultivated in a whey substrate, and blackcurrant juice preparation by-products.

Inhibition Zones of the Growth of Pathogenic Bacteria by *Artemisia absinthium* Water Extract, mm														
**Pathogenic and Opportunistic Strains**														
**1**	**2**	**3**	**4**	**5**	**6**	**7**	**8**	**9**	**10**	**11**	**12**	**13**	**14**	**15**
-	-	-	-	-	-	-	-	-	-	-	-	-	-	20.0 ± 4.1
**Inhibition Zones of the Growth of Pathogenic Bacteria by LUHS245, mm**														
**1**	**2**	**3**	**4**	**5**	**6**	**7**	**8**	**9**	**10**	**11**	**12**	**13**	**14**	**15**
14.1 ± 0.2 ^f^	13.3 ± 0.2 ^e^	16.0 ± 0.2 ^d^	15.3 ± 0.4 ^e^	15.3 ± 0.2 ^d^	16.3 ±0.5 ^g^	16.1 ± 0.3 ^e^	20.0 ± 0.5 ^e^	21.5 ± 0.3 ^h^	-	13.3 ± 0.2 ^b^	14.1 ± 0.3 ^c^	19.5 ± 0.4 ^f^	16.4 ± 0.3 ^c^	22.0 ± 0.2 ^b^
**Inhibition Zones of the Growth of Pathogenic Bacteria by Blackcurrant Juice Preparation By-products, mm**														
-	10.2 ± 0.2 ^a^	16.2 ±0.1 ^d^	14.6 ± 0.1 ^c^	12.2 ± 0.3 ^b^	14.0 ± 0.2 ^c^	12.5 ± 0.1 ^b^	14.3 ± 0.3 ^c^	21.4 ± 0.2 ^h^	20.1 ± 0.1 ^g^	-	10.3 ± 0.2 ^a^	17.0 ± 0.3 ^e^	18.1 ± 0.4 ^f^	30.7 ± 0.5 ^i^

Data expressed as mean values (*n* = 3) ± SD (standard deviation). ^a–i^ Mean values with different letters are significantly different (*p* ≤ 0.05). 1—*Klebsiella pneumoniae;* 2—*Salmonella enterica* 24 SPn06; 3—*Pseudomonas aeruginosa* 17-331; 4—*Acinetobacter baumanni* 17-380; 5—*Proteus mirabilis; 6—*Methicillin-resistant *Staphylococcus aureus* M87fox; 7—*Enterococcus faecalis* 86; 8—*Enterococcus faecium* 103; 9—*Bacillus cereus* 18 01; 10—*Streptococcus mutans*; 11—*Enterobacter cloacae;* 12—*Citrobacter freundii;* 13—*Staphylococcus epidermidis;* 14—*Staphylococcus haemolyticus;* 15—*Pasteurella multocida.*

**Table 2:** Antimicrobial activities of the *Artemisia absinthium* EO (concentration 0.1%) and *Artemisia absinthium* water extract (concentrations 10%, 20%, 30%) against pathogenic opportunistic microorganisms in liquid medium (+ indicated pathogen growth; − indicated that pathogen growth was not observed).

	Pathogenic and Opportunistic Strains														
	1	2	3	4	5	6	7	8	9	10	11	12	13	14	15
A EO	+	+	+	+	+	-	+	-	-	-	+	+	-	+	-
Aex 10%	+	+	+	+	+	+	+	+	+	+	+	+	+	+	+
Aex 20%	+	+	+	+	+	+	+	+	+	+	+	+	+	+	+
Aex 30%	+	+	+	+	+	+	+	+	+	+	+	+	+	+	+
Pathogen control	+	+	+	+	+	+	+	+	+	+	+	+	+	+	+

A—Artemisia absinthium; ex—water extract; EO—essential oil. 1—Klebsiella pneumoniae; 2—Salmonella enterica 24 SPn06; 3—Pseudomonas aeruginosa 17-331; 4—Acinetobacter baumanni 17-380; 5—Proteus mirabilis; 6—Methicillin-resistant Staphylococcus aureus M87fox; 7—Enterococcus faecalis 86; 8—Enterococcus faecium 103; 9—Bacillus cereus 18 01; 10—Streptococcus mutans; 11—Enterobacter cloacae; 12—Citrobacter freundii; 13—Staphylococcus epidermidis; 14—Staphylococcus haemolyticus; 15—Pasteurella multocida.

**Table 3:** *Lactobacillus uvarum* LUHS245 count in whey enriched with different quantities of glucose, yeast extract, and saccharose.

Sugars		*Lactobacillus uvarum* LUHS245		
		Incubation, h		
		12 h	24 h	48 h
		Lactic Acid Bacteria Count, log_10_ CFU mL^−1^		
		6.28 ± 0.16 ^a^	5.34 ± 0.14 ^a^	4.29 ± 0.14 ^a^
Glucose	0.5	6.98 ± 0.09 ^c^	6.91 ± 0.12 ^b,c^	6.92 ± 0.11 ^d^
	1.0	6.39 ± 0.11 ^a,b^	6.73 ± 0.14 ^b,c^	7.06 ± 0.12 ^e^
	1.5	6.42 ± 0.12 ^a,b^	6.40 ± 0.16 ^b^	6.35 ± 0.14 ^b^
	2.0	6.41 ± 0.14 ^a,b^	6.75 ± 0.12 ^b,c^	7.12 ± 0.09 ^e^
	2.5	6.20 ± 0.12 ^a^	7.40 ± 0.11 ^d^	7.21 ± 0.13 ^e^
	3.0	6.83 ± 0.11 ^c^	6.63 ± 0.12 ^b,c^	6.60 ± 0.14 ^c^
	3.5	6.20 ± 0.12 ^a^	6.52 ± 0.13 ^b^	6.69 ± 0.10 ^c^
	4.0	6.46 ± 0.12 ^a,b^	6.61 ± 0.10 ^b,c^	6.80 ± 0.14 ^c^
	4.5	7.22 ± 0.15 ^d^	6.52 ± 0.14 ^b^	6.45 ± 0.11 ^b^
	5.0	6.34 ± 0.12 ^a^	6.54 ± 0.12 ^b^	6.65 ± 0.09 ^c^
Yeast Extract	0.5	7.23 ± 0.14 ^b^	6.63 ± 0.11 ^b^	7.21 ± 0.10 ^d^
	1.0	7.28 ± 0.13 ^b^	7.22 ± 0.12 ^c^	6.56 ± 0.12 ^b^
	1.5	7.30 ± 0.13 ^b^	7.69 ± 0.13 ^d^	7.08 ± 0.11 ^c^
	2.0	7.53 ± 0.15 ^c^	8.11 ± 0.14 ^d^	7.01 ± 0.14 ^c^
	2.5	7.54 ± 0.14 ^c^	7.69 ± 0.12 ^d^	7.30 ± 0.10 ^e^
	3.0	7.83 ± 0.18 ^d^	7.85 ± 0.10 ^d^	7.73 ± 0.10 ^f^
	3.5	7.90 ± 0.13 ^d^	7.93 ± 0.14 ^d^	7.85 ± 0.10 ^f^
	4.0	7.94 ± 0.12 ^d^	7.92 ± 0.17 ^d^	7.90 ± 0.11 ^f^
	4.5	7.92 ± 0.13 ^d^	7.94 ± 0.12 ^d^	7.85 ± 0.12 ^f^
	5.0	7.90 ± 0.14 ^d^	8.06 ± 0.14 ^d^	7.84 ± 0.11 ^f^
Saccharose	0.5	6.82 ± 0.10 ^f^	7.83 ± 0.12 ^c^	8.21 ± 0.13 ^c^
	1.0	6.19 ± 0.11 ^e^	7.84 ± 0.11 ^c^	7.84 ± 0.12 ^b^
	1.5	6.09 ± 0.12 ^d^	7.71 ± 0.13 ^c^	7.90 ± 0.10 ^b^
	2.0	6.08 ± 0.14 ^d^	7.75 ± 0.14 ^c^	7.81 ± 0.14 ^b^
	2.5	6.07 ± 0.10 ^d^	7.76 ± 0.12 ^c^	7.94 ± 0.12 ^b^
	3.0	5.60 ± 0.15 ^c^	5.11 ± 0.10 ^a^	7.51 ± 0.10 ^a^
	3.5	5.41 ± 0.13 ^b^	5.52 ± 0.12 ^b^	7.49 ± 0.13 ^a^
	4.0	5.10 ± 0.12 ^a^	5.10 ± 0.10 ^a^	7.45 ± 0.14 ^a^
	4.5	5.13 ± 0.14 ^a^	5.11 ± 0.11 ^a^	7.42 ± 0.11 ^a^
	5.0	5.11 ± 0.10 ^a^	5.39 ± 0.13 ^b^	7.40 ± 0.09 ^a^

Values are mean ± SD of three replicate analyses (*n* = 3). ^a–f^ Mean values within a row (comparison of the different LAB count: within the same LAB, at the different sugars’ concentrations. Samples enriched with glucose and yeast extract were compared with control whey samples; samples enriched with saccharose were compared between each other) with different letters are significantly different.

**Table 4:** Nutraceuticals colour coordinates (NBS), texture (mJ), total phenolic compounds content (mg 100g^−1^ d.m.), antioxidant activity (%), and overall acceptability (from 140 – extremely like to 0 – extremely dislike).

Formulations of the Different Layers of Nutraceutical	Colour Coordinates, NBS					Texture Hardness, mJ	TPC Content, mg 100 g^−1^ d.m.	Antioxidant Activity, %	Overall Acceptability
	L*	a*			b*				
Aex+Ag	24.29 ± 0.12 ^b^	1.37 ± 0.10 ^b^			7.94 ± 0.23 ^a^	0.70 ± 0.09 ^c^	206.1 ± 5.6 ^a^	42.68 ± 2.4 ^b^	40.6 ± 5.3 ^a^
Aex+G	57.78 ± 0.10 ^f^	1.59 ± 0.14 ^b^			25.39 ± 0.19 ^c^	0.85 ± 0.11 ^c^	195.6 ± 6.2 ^a^	36.47 ± 3.1 ^a^	38.0 ± 4.4 ^a^
Aex+Ag+245	48.72 ± 0.21 ^d^	0.77 ± 0.09 ^a^			20.94 ± 0.17 ^b^	0.30 ± 0.06 ^b^	219.0 ± 7.1 ^b^	78.35 ± 4.8 ^d^	52.8 ± 6.4 ^a,b^
Aex+G+245	54.04 ± 0.13 ^e^	4.69 ± 0.15 ^d^			25.43 ± 0.21 ^c^	0.23 ± 0.05 ^a^	230.1 ± 4.9 ^c^	72.74 ± 3.9 ^c^	43.0 ± 5.0 ^a^
Aex+Ag+245+Bl	23.52 ± 0.20 ^a^	11.15 ± 0.11 ^f^			7.75 ± 0.16 ^a^	1.10 ± 0.09 ^d^	270.3 ± 6.8 ^e^	81.96 ± 4.5 ^d^	125.4 ± 6.1 ^c,d^
Aex+G+245+Bl	22.11 ± 0.17 ^a^	10.19 ± 0.26 ^e^			7.26 ± 0.14 ^a^	0.20 ± 0.04 ^a^	264.6 ± 5.4 ^d^	75.15 ± 3.9 ^d^	103.2 ± 7.9 ^c^
First Layer (Aex+Ag and Aex+G) Tests of Between-Subjects Effects									
Source	Dependent Variable		Type III Sum of Squares			df	Mean Square	F	Sig.
Agar × Gelatin	L*		1682.370			1	1682.370	137899.193	0.0001
	a*		0.073			1	0.073	4.905	0.091
	b*		456.754			1	456.754	10264.129	0.0001
	Texture, mJ		0.034			1	0.034	3.342	0.142
	TPC content, mg 100 g^−1^ d.m.		165.375			1	165.375	4.739	0.095
	Antioxidant activity, %		57.846			1	57.846	7.527	0.052
	Overall acceptability		10.140			1	10.140	0.427	0.549
First and second layer (Aex+Ag+245 and Aex+Ge+245) Tests of Between-Subjects Effects									
Agar × Gelatin	L*		42.454			1	42.454	1391.921	0.0001
	a*		23.050			1	23.050	1506.510	0.0001
	b*		30.240			1	30.240	828.497	0.0001
	Texture, mJ		0.007			1	0.007	2.410	0.196
	TPC content, mg 100 g^−1^ d.m.		184.815			1	184.815	4.967	0.090
	Antioxidant activity, %		47.208			1	47.208	2.468	0.191
	Overall acceptability		144.060			1	144.060	4.368	0.105
Whole nutraceutical formulation (Aex+Ag+245+Bl and Aex+G+245+Bl) Tests of Between-Subjects Effects									
Agar × Gelatin	L*			2.982		1	2.982	86.565	0.001
	a*			1.382		1	1.382	34.690	0.004
	b*			0.360		1	0.360	15.936	0.016
	Texture, mJ			1.215		1	1.215	250.515	0.0001
	TPC content, mg 100 g^−1^ d.m.			48.735		1	48.735	1.293	0.319
	Antioxidant activity, %			69.564		1	69.564	3.924	0.119
	Overall acceptability			223.260		1	223.260	4.482	0.102

Aex—*Artemisia absinthium* essential oil; Ag—agar; G—gelatin; 245—*Lactobacillus uvarum* LUHS245 strains cultivated in dairy by-products; Bl—blackcurrant juice preparation by-products; TPC—total phenolic compounds content; L*—lightness; a*—redness; b*—yellowness. Data expressed as means (*n* = 3) ± SD; SD*—*standard deviation. ^a–f^ Mean values with different letters are significantly different (*p* ≤ 0.05).
